# Evolving Dairy Cattle Systems in Chile: Structural Shifts and Adaptation Strategies

**DOI:** 10.3390/ani14152245

**Published:** 2024-08-01

**Authors:** Paula Toro-Mujica, Raúl Vera-Infanzón

**Affiliations:** 1 Instituto de Ciencias Agroalimentarias, Animales y Ambientales, Universidad de O’Higgins, San Fernando 3070000, Chile; 2 Independent Researcher, 2 Norte 443, Viña del Mar 2520274, Chile; rvi.2005@gmail.com

**Keywords:** typology, dual purpose, geographical mapping

## Abstract

**Simple Summary:**

This study explores the evolution of Chile’s dairy cattle systems from 1997 to 2021. It reveals a notable decline in both the number of dairy farms and cattle stocks, with southern farms gaining prominence. There is a clear trend towards intensification characterized by increased stocking rates and larger herd sizes. Furthermore, there has been a shift in grassland utilization, from natural pastures to improved and sown pastures. It underscores regional disparities and offers insights into the challenges and opportunities facing Chile’s dairy sector.

**Abstract:**

This study examines the structural and geographical changes in Chile’s dairy cattle systems from 1997 to 2021, using data from agricultural censuses. It focuses on variables like animal stocks, herd sizes, pasture utilization, and stocking rates, analyzed through descriptive statistics, multivariable analysis, and geographical mapping. The findings show a significant decrease in dairy farms (−69%) and dairy cattle stocks (−25.4%), with regional disparities: southern farms increased in importance while central farms declined. There is a trend towards intensification, with higher stocking rates and larger herd sizes. Grassland utilization shifted, with a decrease in natural pastures and an increase in improved and sown pastures. The study identifies four typological groups based on stocking rates, dimensions, and pasture use, reflecting distinct evolutionary paths influenced by climate change, land use, economic dynamics, and technology. Geographical analyses highlight regional variations. This research provides insights into the dynamics of Chile’s dairy farming sector and its sustainability challenges and opportunities amidst changing environmental and socioeconomic conditions.

## 1. Introduction

On a global scale, animal production is scrutinized from various perspectives, including the degradation of natural resources, deforestation, animal welfare, food safety, and the emission of greenhouse gases [1,2,3]. Livestock production contributes 14.5% of global greenhouse gas emissions, exceeding 7.1 gigatons of CO_2-equivalent_ annually. Specifically, cattle milk production accounts for 20% of the sector’s emissions [4], making it a significant contributor to global warming. Furthermore, the rise of ecological, vegetarian, vegan, and animal rights movements has amplified societal interest in understanding production systems. These movements aim to enhance animal welfare, conserve natural resources, and emphasize the importance of the origins and methods of food production [5,6,7]. Consequently, these factors, alongside traditional production factors, shape the trajectory of production systems.

Ma et al. [8] in China highlight the global trend of decreasing numbers of dairy cows in farms with fewer than 10 cows and increasing numbers in farms with herds of over 500 cows. Zorn and Zimmert [9] associate the likelihood of Swiss dairy farms exiting the industry with herd size, noting that the probability of exit decreases as herd size increases. The prevalence of larger herds, driven by the pursuit of economies of scale, is supported by global findings in dairy production. Roche et al. [10] describe key discoveries in dairy production over the past 100 years that have driven changes in milk production systems. For example, in infrastructure, innovations include the creation of electric fencing, the herringbone milking parlor, and rotary or carousel dairies. In pasture management, advances involve using sward height and mass measurements to determine optimal grazing periods, improving soil fertilization plans, proposing morphological stages to select optimal grazing points, and defining seasonal rotation plans. In reproduction, the use of artificial insemination, sexed semen, estrus detection and synchronization systems, and genetic improvement programs have been pivotal. In nutrition, the adoption of transition diets, precalving supplementary feed, and the management of milk fever have made significant impacts.

Added to these advances, among traditional production factors, economic considerations such as production costs, scale, profitability, and the opportunity cost of local labor remain pivotal. Irrespective of the degree of intensification of animal production, feed costs persist as the primary expense making it a key determinant for the adaptation or discontinuation of production systems [11,12]. 

The utilization of natural or improved grasslands as a feed source in extensive systems reduces the purchase of external inputs and ensures stability in feed costs, providing greater system flexibility amidst market uncertainties [13,14]. However, the use given to a particular territory is conditioned by various factors, including climate, availability of irrigation, investment capacity, and, significantly, the relative profitability of the activity [15].

Chile has a diversity of climates including a desert climate in the north, arid and semiarid conditions in the central zone, temperate and rainy in the south, and tundra in the extreme south of the country [16]. Nevertheless, the impacts of climate change have reshaped the climatic map, leading to reduced precipitation and higher temperatures nationwide, consequently expanding the arid areas [17]. In this scenario, when irrigation is possible, and the climate allows for it, territories tend to be allocated to more profitable activities. Fruit and wine production are highly profitable, and the expansion of the territory induced by climate change and improved irrigation efficiency has displaced livestock production to the country’s southern regions. Consequently, livestock production persists only on farms capable of adapting to remain competitive in profitability. However, adaptation is a process necessitating structural changes in livestock production systems, thereby influencing their temporal evolution. 

The evolution of production systems for different species has been studied through different methodologies of data collection and subsequent analysis. Data collection methods include surveys of a representative sample of farms in successive periods [14,18], the use of official livestock surveys [19], and the use of census data [20] applied to specific geographical areas or at the country level. Data analysis entails univariate techniques such as frequency tables, ANOVA, Kruskal–Wallis, and chi-square tests tailored to the variable types as well as multivariate methods like multiple correspondence analysis (for categorical variables) and principal component analysis (for quantitative variables) to identify associations and patterns [21]. Recent applications of these methodologies in animal production include Rjili et al. [14], who analyzed the changes in farms within arid rangelands of south Tunisia from 2004 to 2019; Ibidhi et al. [22], who identified watering in sheep production systems; and Bánkuti et al. [23], who assessed the sustainability of dairy farms classifying farms into high, intermediate, and low sustainability groups.

According to the FAO [24], the global bovine population has increased by approximately 16% in the last 20 years, from 1320 million heads in 2000 to 1529 million in 2021, associated with a 52.4% increase in dairy bovine production. During this same period, Chile experienced an increase in milk production of 14%, coupled with a decrease in the bovine population from 4.068 million to 3.038 million. These changes are indicative of shifts in production systems. Thus, this work aims to analyze the geographical and temporal evolution of the dairy cattle population in Chile and identify the continuity strategies adopted by their production systems.

## 2. Materials and Methods

### 2.1. Study Area

**Figure 1:**
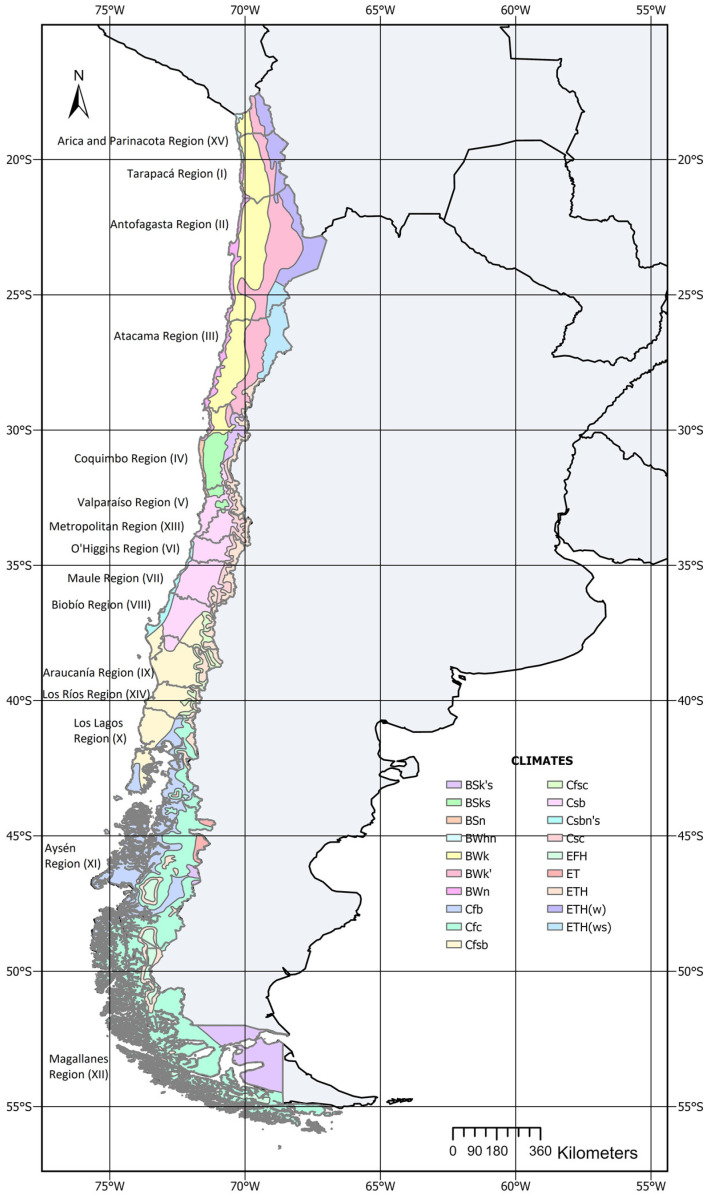
Chile climates and regions.

The study area encompasses Chile, a country with a length of 4270 km, an average width of 180 km, and a surface area of 756,945 km^2^. Until 2017, Chile was divided into 15 regions. Following the division of the Biobío region, the country now has 16 regions, each with a diverse climate [16] (Figure 1).

### 2.2. Data Collection

Data were sourced from the 1997, 2007, and 2021 agricultural censuses. The 1997 and 2007 microdata databases were obtained from the National Statistics Institute under Law 20285 [25]. These were provided in digital format and stored in Access^®^. The 2021 Census data were acquired directly from the National Statistics Institute website in CSV format [26]. Each database contained sheets classified by agricultural, livestock, and forestry activities, with rows representing farms and columns representing variables. Farms had unique identifiers for data consolidation. Data preparation included (i) selecting dairy cattle farms; (ii) matching these farms with data on crops, fruits, pastures, forestry species, and land area; (iii) selecting variables present in all three censuses; and (iv) processing variables to calculate resource use indicators. This step was necessary due to differences in census questionnaires across years. A global database was created with indicators for each year, region, and farm. 

### 2.3. Data Analysis

Descriptive statistics for dairy cattle stocks were obtained at regional and national levels, with maps showing the evolution of cattle populations and farms, categorized by cow and total cattle numbers. Data analysis included chi-square tests, ANOVA, and mean comparison tests.

A multivariable analysis of the 1997 census data followed Toro Mujica et al. [20], involving variable selection, factorial analysis, and cluster analysis. Variables with a coefficient of variation over 50% were selected, and correlated variables were excluded. Principal component analysis (PCA) retained components with eigenvalues over 1. Outlier farms were removed using factorial scores, and variable suitability was assessed with the Kaiser–Meyer–Olkin measure and Bartlett’s test. The partitioning around medoids (PAM) method was used for clustering, with the number of clusters determined by the silhouette method [27]. Factorial scores for 2007 and 2021 farms were calculated using equations from the factorial analysis, and group membership was determined through discriminant analysis [20]. Linear discriminant analysis (LDA) and quadratic discriminant analysis (QDA) were evaluated, selecting the model with the highest correct classification rate.

Group evolution across census years was compared using ANOVA and multiple mean comparison tests. Analyses were performed with INFOSTAT and RStudio. The experimental design included unifactorial and factorial experiments to evaluate the effects of year, region, group, animal load, and herd size on census variables.

## 3. Results and Discussion

### 3.1. Description of the Bovine Population and National Distribution

#### 3.1.1. Farms Number

Between 1997 and 2021, the number of dairy cattle farms in Chile decreased by 69%, from 46,900 in 1997 to 14,222 in 2021 (Table 1). This trend is consistent with other South American countries, such as Argentina, which saw a 41% reduction between 1998 and 2018 [28], and southern Brazil, with a 32% decrease [29].

**Figure 2:**
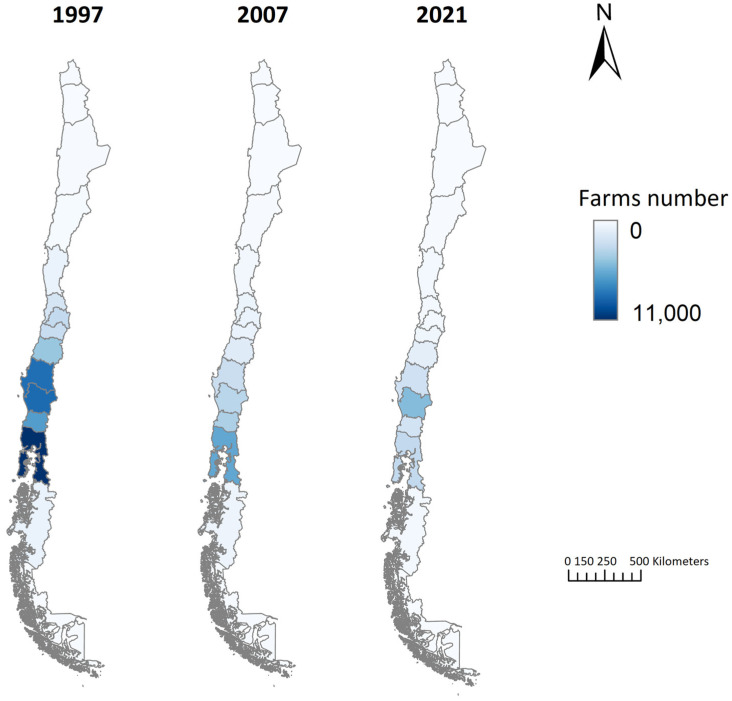
Spatial and temporal distribution of dairy cattle farms in Chile.

In 1997, 56.5% of dairy farms were located in the southern zone of Chile (Regions IX, X, XI, XII, and XIV). This percentage increased to 68.0% in 2007 and remained stable at 68.6% in 2021 (Figure 2). Conversely, the central zone (Regions V, XIII, VI, VII, and VIII) saw a significant decline, from 41.7% in 1997 to 29.2% in 2021. The Metropolitan region (XIII) experienced the most dramatic reduction, with a 90.6% decrease in farms.

This geographical concentration of dairy farms is a global trend. For example, over 52% of Brazil’s dairy farms are in the southern zone [30]. The decline in the number of farms, alongside increased herd size and productivity per animal and hectare, aligns with global trends [31,32].

#### 3.1.2. Animal Stocks

**Figure 3:**
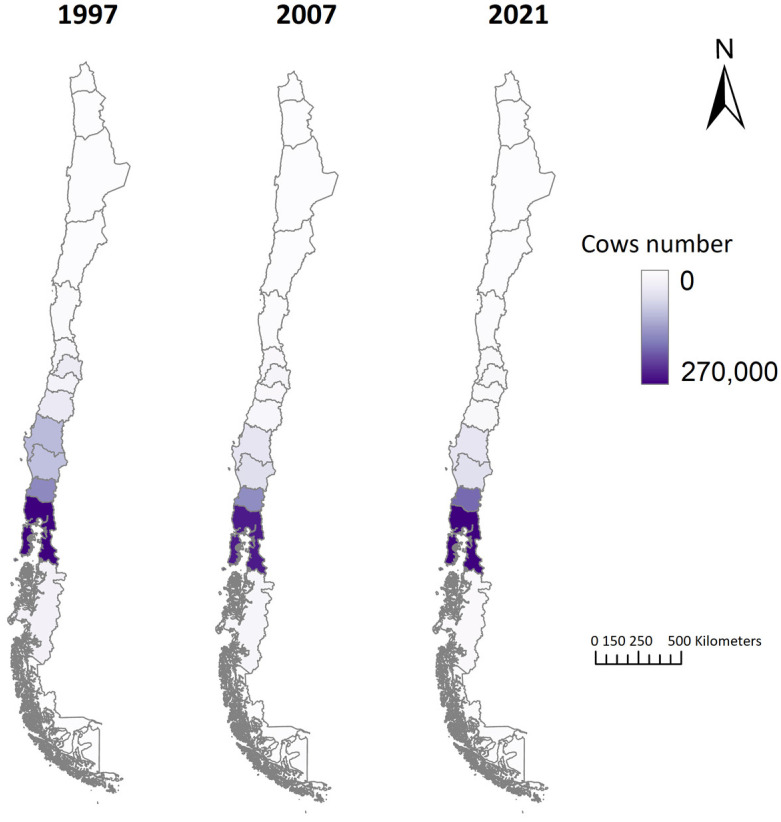
Spatial and temporal distribution of dairy cattle in Chile.

Chile’s stock of purebred and dual-purpose dairy cows fell by 25.4% between 1997 and 2007 and by 13.3% between 2007 and 2021, decreasing from 761,860 animals in 1997 to 492,760 in 2021 (Table 2). In 1997, the southern zone held 70.7% of the dairy cattle, increasing to 85.1% by 2021. Conversely, the central zone experienced a 67.5% decrease in dairy cattle stocks from 1997 to 2021 (Figure 3).

The worldwide cattle population increased by 12.5% between 1997 and 2021, although 42% of the countries experienced a decrease. Europe had the most significant decline, with a 30% reduction in stocks across 33 countries. This trend began in the 19th century due to industrialization and continued after World War II, intensifying with increased productivity and agricultural intensification, and more recently, changes in the Common Agricultural Policy [33,34]. In contrast, the Americas saw a 17.6% increase in cattle stocks, although 44% of countries, including the United States, Canada, Ecuador, and Chile, experienced declines [24].

#### 3.1.3. Cattle Stocking Rate and Herd Size

Herd sizes in Chile vary widely, ranging from a single cow on many farms to one farm with 36,231 cows, reflecting the coexistence of family and commercial farms. This disparity is typical in livestock systems. The trend of farm intensification and growth has persisted for decades, driven by the need to reduce costs and increase production efficiency [11,35]. Between 1997 and 2021, the average stocking rate increased by 15.8%, with significant regional variations. Eight out of fifteen regions showed statistically significant changes in stocking rates, particularly in Central Chile (Regions IV to VII), where rates reached levels typical of semi-intensive systems.

Marín et al. [36] point out that land use and cover change are driven by the interaction of ecological, geographical, economic, and social factors that determine the trajectories of landscape development. Thus, farm stocking rates are influenced by forage availability and by the cost and availability of purchased inputs [37]. The profitability of other productive activities, such as fruit production, affects land values and available forage crops. In Central Chile, fruit production expanded by 15.6% from 2007 to 2021, covering 381,691 hectares [38]. Southward, the climate becomes colder and rainier, favoring pastoral systems with lower stocking rates (Figure 1). However, in the Magallanes region (XII), the cold, rainy climate necessitates supplementation and confinement during winter, increasing stocking rates, which are similar to those of year-round pasture-based dairy systems in Uruguay [39,40].

Increasing herd size aims to enhance production efficiency through economies of scale [41]. This growth has been recorded in six regions, with rising land prices in Regions V, XIII, and VI contrasting with the southern regions where pastoral systems remain prevalent [19]. 

#### 3.1.4. Grassland Use

Grasslands in Chile were classified into natural, improved, and sown categories in all three censuses. Improved grassland are areas of land where management practices have been implemented to increase the productivity and quality of the existing grass. These practices may include no-till seeding, fertilization, weed control, and other management techniques to optimize the use of the pasture. Sown grassland are areas of land where vegetation cover has been deliberately established by sowing seeds of specific grasses or legumes. These grasslands are designed and managed to maximize forage production for livestock.

The relative importance and variation of each type depended on the region (*p* < 0.01) and census year (*p* < 0.01). Nationally, natural grassland consistently represented the largest land use (Table 3). Regions XIII, VI, and VII saw significant increases in natural grassland, contrasting with declines in improved grassland, because the cost of improvements exceeds the expected returns. Rising fertilizer prices and decreased irrigated areas [42] may explain this trend. Additionally, reduced irrigated areas have necessitated more efficient water usage, favoring irrigated sown grassland.

In the northern regions (XV, I, II, and III), natural and improved grasslands are less significant compared to sown grassland. The decreased precipitation of Regions XIII and VI [43] has led to increasing investments in irrigation and the establishment of irrigated sown pastures. This adaptation reflects strategies to cope with climate change, such as improving irrigation systems and changing crops [44]. In contrast, the central-southern and southern regions (XIV, X, XI, and XII) have climates (Cfsb, Cfc, and Csb) conducive to long growing seasons, making natural and improved grasslands comparable in yield to sown grasslands. However, the shortening of the rainy season and increasing temperatures associated with climate change are affecting grassland management strategies [45].

### 3.2. Categorization of Farms

#### 3.2.1. Herd Size

Dairy cattle stocks were categorized into six groups based on cow livestock units (CLUs) (Table 4). The first range (≤10 CLU) represented over 63% of total farms in all three census years, similar to findings by Mendoca et al. [46], who reported that over 60% of Brazilian dairy farming involves family farmers. The chi-square test showed a dependence between census year and farm proportion in each category (X^2^ = 1384.3, 10 df, *p* < 0.01). Farms with fewer than 500 cows decreased by over 70% from 1997 to 2021, while those with more than 1000 cows increased by 555.6%.

This decrease in dairy farms aligns with trends in Canada, the USA, and China, as noted by Tonet et al. [29], due to institutional and market instability. Brito et al. [47] attribute this to increased milk production per animal, highlighting that around 95% of high-yielding dairy cows globally belong to three breeds: Holstein, Jersey, and Brown Swiss. In Chile, Holstein Friesian cows dominate the specialized dairy system, while breeds like Overo Negro, Overo Colorado, Jersey, and Normando are common in other systems.

Census year significantly affected stocking rates (*p* < 0.01) and herd size (*p* < 0.01), according to a multifactorial ANOVA. Stocking rates increased in three of the six categories (Table 5). Macdonald et al. [48] noted a positive correlation between milk production per hectare and stocking rate in various experiments but cautioned against generalization due to differences in experiment duration, treatments, and feed use.

#### 3.2.2. Stocking Rates

Farms were grouped into six stocking rate ranges (bovine livestock units, BLU/ha; Table 6). Herd sizes varied widely within these ranges, generally increasing over time across all categories. However, the number of farms decreased as the stocking rate increased (X^2^, 1090.3, 12 df, *p* < 0.01), except in the 5–10 LU/ha range. Notably, the 2–3 and 3–4 LU/ha groups experienced the smallest declines in farm numbers (50.5% and 51.8%, respectively). This persistence is due to specialization in dairy breeds, which require concentrated diets to maximize productivity [47]. There is also growing interest in grazing systems due to lower milk prices, higher production costs, and concerns about the environmental and animal welfare impacts of intensive dairying [4,48].

### 3.3. Relationship between Categories of Bovine Stocking Rates and Types of Pasture

The percentage of natural pastures increased significantly between census years in the 2–3 and 3–4 BLU/ha categories (Table 7). Both stocking rates (*p* < 0.01) and census year (*p* < 0.01) significantly affected the percentage of natural pastures, with the highest percentages found at stocking rates below 3 BLU/ha in all census years (Table 7). Macdonald et al. [49] noted that the economic optimum stocking rates for grazing dairy systems depend on pasture production potential, purchased feed, and cow weight. Farms with lower BLU/ha typically rely on natural pastures, which are less productive than sown or improved pastures within the same agro-climatic zone.

Intensification can be achieved by fertilizing pastures, reducing grazing periods, and incorporating more concentrated feed [50]. While both the stocking rate and census year significantly affected the percentage of improved pasture (*p* < 0.01), there was no clear trend of increase or decrease as the stocking rate increased. The highest percentages of improved pasture were found in the 1–2 and 2–3 BLU/ha ranges (Table 8). Similarly, the stocking rate and census year significantly impacted the percentage of sown pasture (*p* < 0.01). At stocking rates above 4 BLU/ha, no significant changes were observed in the percentage of sown pasture, while lower stocking levels saw a slight decrease (Table 9).

### 3.4. Multivariable Analysis

#### Variable Selection

Sixteen variables were obtained from census data (Table 10). Only the relative presence of cows in the herd (CLUB) and on the farm (CLUT) had a coefficient of variation (CV) below 50%. These two variables were retained given their limited variation (0–100%) and importance in defining system typology. Variables with low correlations (r < 0.3) including the relative presence of forest species (FPP), orchards (OPP), crops (CPP), and farm area were discarded. The farm stocking rate variable was also discarded due to its strong association with the bovine stocking rate variable (BSR; r > 0.9).

### 3.5. Factor Analysis

The initial factor analysis identified six outlier farms, which were removed. After their removal, the factor analysis showed a KMO (Kaiser–Meyer–Olkin) measure of 0.7, deemed adequate [51]. Bartlett’s test of sphericity was significant (*p* < 0.01). Four principal components were identified, representing 76.7% of the variance (Table 11). Three to five principal components (PC) explaining over 50% of the variance are typical in developing countries typologies for livestock farms [14,52,53,54].

The first principal component (PC1) represented 34.4% of the data variance. It was associated with dimension, incorporating the variables number of bulls, cows, livestock unit (CLU), size of the herd (SH), and total livestock unit (TLU; Table 12). Similar associations between dimensional variables and PC1 have been observed in various livestock systems studies. For instance, Gökdai et al. [53] found that PC1 was significantly associated with production level, input purchases, and forage consumption in dairy goats. PC2 was related to the specificity of dairy and cattle production, incorporating the relationships between CLU and SH (CLUB) and between CLU and TLU. PC3 was linked to forage resource use, showing a negative relationship with the use of natural pasture. PC4 was associated with the relative presence of sown pasture and the bovine stocking rate, indicating that more intensive production systems had higher PC4 scores (Table 12). Toro-Mujica et al. [19] also found a relationship between PC4 and the importance of sown pasture in dairy cattle studies in southern Chile, although stocking rate was associated with PC3 in their research.

### 3.6. Cluster Analysis

**Figure 4:**
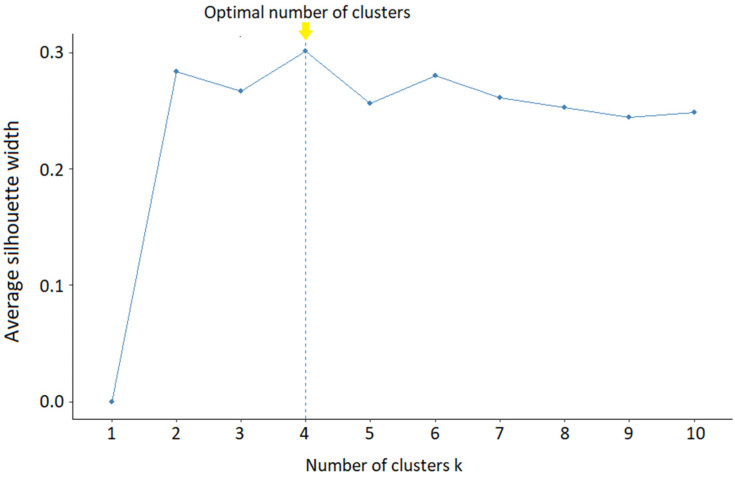
Selection of the number of clusters through the silhouette coefficient.

The maximum silhouette coefficient was 0.301, a value obtained for grouping four clusters, which was determined through Figure 4 and considered acceptable [55].

Studies on the typology of dairy cattle systems with various objectives and clustering methodologies have frequently selected three to six groups [19,23,54,56].

### 3.7. Discriminant Analysis and Group Definition

The LDA and QDA methodologies correctly classified 95.8% and 95.5% of the farms, respectively. Due to its higher accuracy, the LDA methodology was used to predict the group membership of farms in the 2007 and 2021 censuses. In the 1997 census, 69% of the farms were classified in Groups I and II, with similar percentages (Table 13). In the following two censuses, a significant increase in the relative importance of Group I was observed, reaching 50.1% of the existing farms in 2021. All groups showed a decrease in the number of farms. However, while the decrease in farms exceeded 75% in Groups II to IV, it was 56% in Group I (Table 13).

**Figure 5:**
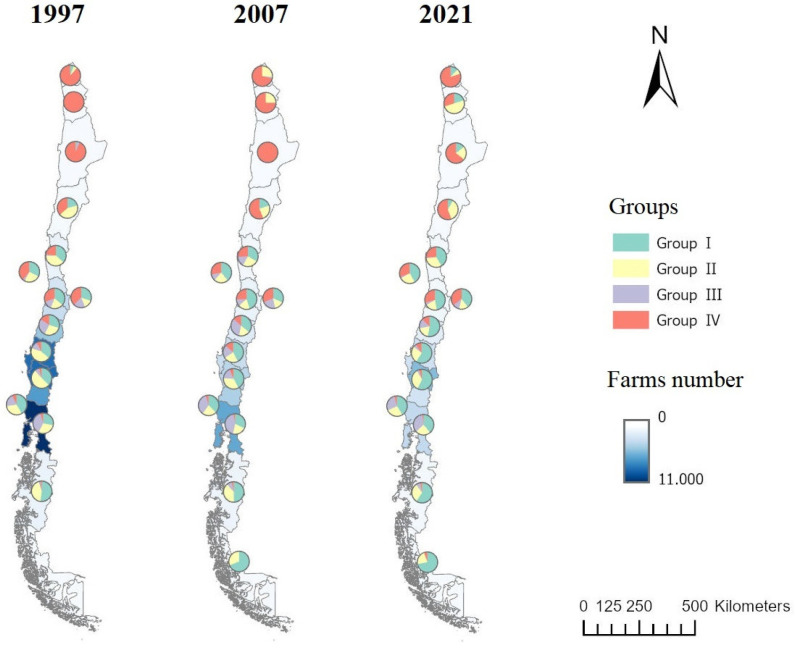
Distribution and geographic evolution of groups.

The identified groups exhibited high variability in dimensional variables (CLU and farm area), indicating that it is possible to find the same production system at different scales. Analyzing the average values of the variables, the groups were defined as follows:

Group I: Extensive System. These correspond to small- to medium-sized farms with low stocking rates, primarily based on using natural pasture for feeding. In this group, the highest percentage of bovine animal units corresponds to cows (73%), evidencing a tendency towards dairy activity. Farms of this group increase in relative importance as one moves south of Region II (Figure 5), along with an increase in herd sizes. Although the presence of dairy or dual-purpose cattle farms in Region XI is scarce, most of the existing ones belong to this group. In this region, properties are large, and naturalized pasture has replaced the territory historically occupied by native forests [57].

Group II: Dual-purpose extensive system. Based on low bovine stoking rates, the lower CLUB and CLUT (Table 14) correspond to a less specialized system, generally with dual-purpose cattle breeds carrying calf rearing. These farms are usually family farms, but commercial farms can also be found, considering the wide variability in farm area and herd size. Along with Group I, these were the most abundant systems throughout the country, although a significantly higher presence was observed in Regions VIII and IX (Figure 5).

Group III: Specialized semi-intensive system. Larger bovine and total stocking rate and herd size characterized this group. The increases in stocking rate were linked to the change in the type of pasture, shifting from natural to improved pasture. In Regions XIV and X, the presence of this type of farm is abundant, mainly due to the climate, which favors grazing naturalized pastures that yield 6 to 8 tons of DM/ha [58].

Group IV: Intensive system. Unlike the previous groups, the bovine stocking rate in this group indicates intensive animal management maintained by sown pastures and complementary crops. These types of farms are common in the northern and central regions of the country, where intensification is driven by land cost and low precipitation. 

### 3.8. Group Evolution

#### 3.8.1. Structural Changes

Structural changes were found in most of the analyzed variables in the four defined groups (Table 15). The main changes in each group are described below.

Herd size increased in Group I, together with specialization in both cattle and dairy farming (CLUB and CLUT), which resulted from increasing the stocking rate, decreasing farm area, and the purchase of commercial feedstuffs facilitated by lower prices of imported grains relative to local costs [26].

No significant change in herd size was noted in Group II, although there was a significant decrease in farm area during the period 2007–2021. Dual-purpose cattle in this system provide greater economic flexibility to farmers, resulting in increased system stability [59]. The megadroughts experienced by the country over the last decades and shortened growing seasons were probably responsible for the reduction in natural pastures [60].

Group III showed significant differences in all analyzed variables (Table 12). Farms in this group increased their herd size markedly with large between-farm variability. Additionally, greater specialization in dairy activity was observed in this group, with an increase in CLUB. The use of improved pasture showed a slight increase, which supports the increase in stocking rate. 

Group IV showed an increase in herd size, bovine stocking rate, and total stocking similar to Group III, but without a sustained increase in farm area. Natural and sown pastures significantly increased their relative importance (RPNP and RPSP), unlike improved pastures (RPIP), which showed a decrease. The increase in the bovine stocking rate may have been sustained both by the increase in the area of sown pasture and by the purchase of external inputs. Simultaneously, there was an increase in CLUB, indicating an increase in the specialization of dairy cattle.

The increase in the average size of agricultural holdings is a trend observed globally in high-income countries but not in low- and middle-income countries [61]. The present results were consistent with this trend although only Group III increased its farm area. Nevertheless, animal numbers increased in three of the groups without parallel changes in farm size. It is hypothesized that these changes were related to pasture growth, investment possibilities, and input prices. Climate change, with its influence on the pasture growth curve, tends to generate a decrease in the use of natural and improved pastures in drier climates or dryland areas.

#### 3.8.2. Geographic Changes

The relationship between census data and regional distribution of the groups (*p* < 0.01) is shown in Figure 5 (statistics in Appendix A). The relative presence of Groups I and II in the country’s northern zone, which in 1997 did not exceed 4% of farms, rose to 20% and 50%, respectively, in 2021. Between 2007 and 2021, Group III decreased its importance in the central zone of Chile, representing less than 2.5% of farms in Regions IV and V. A decrease in the importance of Group III also occurred in the southern zone, between Regions IX and XI. The relative presence of Group IV tended to decrease in most of the country except in the central zone, where increases of 2 to 4 percentage points were observed.

As Gauly and Ammer [62] point out, climate change poses a challenge for dairy cow production systems due to its effect on animal thermal comfort, pasture availability, and crop growth. Climate change in Chile is one of several factors that has modified and will continue to influence the number of dairy farms, their structure, and geographic location. Regarding thermal comfort, studies conducted in Central [63] and southern Chile [64] have shown that during the summer months, the THI (temperature–humidity index) exceeds 68, a borderline value of heat stress [65]. Emergency and danger THI values have been reported for Regions VII and VIII [63], while alert values in Region IX have also been reported.

Melo and Foster [66] simulated climate change scenarios and projected a decrease in the area of natural pastures, an increase in managed pastures, as well as an increase in the area of alfalfa, which would lead to an intensification of livestock systems.

#### 3.8.3. Future Implications

Various dynamic aspects will influence changes in the dairy sector in the future. Firstly, considering climatic factors, dairy producers will need to adopt advanced precision agriculture and livestock technologies to mitigate the effects of climate change on forage production [67], animal welfare [68], and to ensure the sustainability of their production systems. At this point, training producers in the use of these technologies will be crucial in the future evolution of the sector [69]. In the economic dimension of sustainability, changes in input and product prices, as well as in the demand for dairy products, both nationally and internationally, will influence the economic viability of dairy farms. In the social dimension, regulations and public policies are expected to become stricter in terms of animal welfare practices and environmental sustainability, as well as in the rights of agricultural workers, which could require significant adjustments to current production systems.

In summary, the worldwide dairy sector, including that of Chile, will be conditioned by a combination of climatic, economic, technological, social, and regulatory factors. The ability of producers and their farms to adapt to these dynamic factors and adopt innovative strategies will be crucial for the sustainable and competitive development of the dairy sector in the future.

## 4. Conclusions

Agricultural censuses are vital for assessing changes in the agricultural sector, but extracting specific data for scenarios like dairy farming requires extensive work. Dairy cattle systems involve numerous specific variables, and since censuses cover all agricultural activities, isolating dairy-specific data is complex. Despite this, general dimensional variables can still define dairy cattle production systems when processed and correlated with other variables.

In Chile, the bovine population has undergone significant structural changes over the past two decades. These changes include a decrease in the number of dairy cattle farms, a shift towards larger-scale operations, and changes in geographical distribution. This decline in dairy farms aligns with global trends driven by market instability, increased productivity per animal, and changing agricultural policies. The southern zone of Chile has maintained its dominance in dairy farming, while the central zone, particularly the Metropolitan region (XIII), has seen a decrease in farms. Despite fewer farms, there has been an increase in stocking rates, especially in the central zone, indicating a trend toward intensification and increased production efficiency. Grassland use has also evolved, with variations in pasture types across regions.

Multivariable analysis identified key variables influencing farm typology, such as herd size, stocking rates, and the presence of different types of pastures. Cluster analysis revealed four main dairy production systems, each with unique structural changes and geographic distributions over time. These groups reflect trends toward increased specialization, intensification, and adaptation to environmental and economic conditions. Geographic changes in farm distribution highlight the impact of climate change on dairy production, posing challenges related to thermal comfort, pasture availability, and crop growth.

In summary, Chile’s dairy cattle farming sector has undergone significant transformations due to market dynamics, technological advancements, and environmental pressures. Understanding these changes is crucial for developing strategies for sustainable and resilient dairy production systems in response to evolving challenges.

## Figures and Tables

**Table 1:** Number and percentage of dairy cattle farms in the administrative regions of Chile.

Region	1997		2007		2021	
	N°	%	N°	%	N°	%
XV	35	(0.07%)	42	(0.18%)	25	(0.18%)
I	6	(0.01%)	4	(0.07%)	10	(0.07%)
II	47	(0.1%)	5	(0.1%)	14	(0.1%)
II	76	(0.16%)	25	(0.25%)	36	(0.25%)
IV	696	(1.48%)	269	(1.67%)	238	(1.67%)
V	1771	(3.78%)	634	(1.57%)	223	(1.57%)
XIII	2679	(5.71%)	654	(1.76%)	251	(1.76%)
VI	2549	(5.43%)	728	(2.9%)	413	(2.9%)
VII	4234	(9.03%)	1316	(7.54%)	1073	(7.54%)
VIII	8325	17.75%)	2487	15.38%)	2188	(15.38%)
IX	8490	(18.1%)	3149	(32.9%)	4679	(32.9%)
XIV	6333	(13.5%)	3601	(14.2%)	2019	(14.2%)
X	10,940	(23.33%)	5778	19.74%)	2807	(19.74%)
XI	719	(1.53%)	543	(1.6%)	228	(1.6%)
XII	0	(0.00%)	45	(0.13%)	18	(0.13%)
Total	46,900	100%	19,280	100%	14,222	100%

**Table 2:** Regional and national average bovine stocking.

Region	Stocking Rate (LU/ha)				Herd Size (LU/farm)			
	1997	2007	2021	*p* Value	1997	2007	2021	*p* Value
XV	1.3 ± 1.1 ^a^	1.8 ± 2 ^b^	2.1 ± 2.0 ^b^	<0.01	21.0 ± 17.6 ^a^	15.3 ± 18.5 ^b^	18.9 ± 25.9 ^c^	<0.01
I	13.4 ± 20.0 *	4.5 ± 3.7	3.6 ± 3.1	0.25	4.4 ± 2.7	3.2 ± 1.0	5.2 ± 3.4	0.513
II	1.8 ± 3.0	0.7 ± 0.3	1.2 ± 1.3	0.46	4.8 ± 4.4	4.8 ± 3	4.7 ± 7.5	0.989
III	0.9 ± 1.0	1.8 ± 2.0	1.4 ± 1.5	0.11	26.5 ± 37.1 ^a^	64.3 ± 101.5 ^b^	33.9 ± 72.5 ^ab^	0.03
IV	1.1 ± 1.8 ^a^	2.3 ± 6.6 ^b^	2.5 ± 7.5 ^b^	<0.01	9.9 ± 19.5 ^a^	15.2 ± 44.2 ^b^	9.3 ± 41.5 ^ab^	0.04
V	2.8 ± 6.1	2.6 ± 6.6	3.6 ± 7.4	0.12	13.1 ± 46.9 ^a^	20.3 ± 76 ^a^	57.9 ± 273.7 ^b^	<0.01
XIII	2.3 ± 5.2	2.2 ± 4.4	2.7 ± 6.0	0.40	21.7 ± 78.5 ^a^	37.1 ± 109.7 ^b^	49.4 ± 157.8 ^b^	<0.01
VI	1.8 ± 7.6	1.7 ± 7.1	2.0 ± 5.8	0.82	13.5 ± 49.4 ^a^	16.9 ± 53.5 ^ab^	20.4 ± 79 ^b^	0.03
VII	0.8 ± 1.6 ^a^	1.1 ± 2.0 ^b^	1.2 ± 2.4 ^b^	<0.01	15.6 ± 40.7 ^b^	15.4 ± 32.7 ^b^	11.9 ± 23.1 ^a^	0.01
VIII	0.7 ± 1.2 ^a^	1.1 ± 2.1 ^b^	1.1 ± 2.1 ^b^	<0.01	21.9 ± 71.7 ^a^	30.8 ± 119.5 ^b^	24.7 ± 174.4 ^a^	0.01
IX	0.7 ± 0.9 ^a^	0.8 ± 0.9 ^b^	0.8 ± 1.5 ^b^	<0.01	20.3 ± 64.7 ^b^	30.8 ± 123.8 ^b^	15.1 ± 73.5 ^a^	<0.01
XIV	0.8 ± 0.7	1.0 ± 0.7	1.2 ± 1.0	<0.01	41.7 ± 123.2 ^a^	66.3 ± 201.7 ^b^	104.3 ± 399.1 ^c^	<0.01
X	0.7 ± 0.7 ^a^	0.9 ± 0.9 ^b^	1.0 ± 1.0 ^c^	<0.01	42 ± 190.5 ^a^	67.3 ± 247 ^b^	112.4 ± 993.6 ^c^	<0.01
XI	0.2 ± 0.4 ^a^	0.4 ± 0.7 ^b^	0.5 ± 0.9 ^b^	<0.01	56.7 ± 93.2 ^a^	43.1 ± 53.5 ^b^	33.4 ± 44.6 ^b^	<0.01
XII	-----	0.9 ± 1.8	3.0 ± 10.5	0.19	0.0 ± 0.0	49.2 ± 59.7 ^a^	134.2 ± 194.1 ^b^	<0.01
National	1.0 ± 2.7 ^a^	1.1 ± 2.5 ^b^	1.1 ± 2.4 ^b^	<0.01	27.9 ± 114.8 ^a^	46.8 ± 177.6 ^b^	50.0 ± 477.2 ^b^	<0.01

^a,b,c^ Within rows, averages with different superscript differ significantly. * Very few farms in a desertic region.

**Table 3:** Grassland relative importance by region and census.

Reg.	Natural Grassland (%)				Improved Grassland (%)				Sown Grassland (%)			
	1997	2007	2021	*p* Value	1997	2007	2021	*p* Value	1997	2007	2021	*p* Value
XV	0 ± 0	3.4 ± 13.9	0.3 ± 1.3	0.19	0.4 ± 2.4	2.4 ± 15.4	0 ± 0	0.56	48.3 ± 24.9	50.7 ± 33.9	63.4 ± 31.8	0.142
I	0 ± 0	0 ± 0	0 ± 0	----	0 ± 0	0 ± 0	0 ± 0	---	56.5 ± 33.2 ^b^	25.8 ± 24 ^ab^	9.4 ± 19.4 ^a^	<0.01
II	0.3 ± 1.7 ^a^	0 ± 0 ^a^	11.6 ± 23.7 ^b^	<0.01	2.9 ± 12.7	0 ± 0	0 ± 0	0.61	60.9 ± 23.1	57.9 ± 22.9	36.5 ± 25	<0.01
III	6.7 ± 14.1	9 ± 21.4	3.2 ± 8.7	0.297	0.7 ± 3.6	1 ± 3.5	0 ± 0	0.4	15.2 ± 16 ^a^	33.8 ± 28.5 ^b^	30.4 ± 26.4 ^b^	<0.01
IV	8.9 ± 22.2 ^a^	18.9 ± 28 ^b^	9.8 ± 21.5 ^a^	<0.01	1.6 ± 7.4 ^a^	14.3 ± 27.4 ^b^	0.8 ± 5.8 ^a^	<0.01	10.2 ± 17 ^a^	14.5 ± 23.8 ^b^	12.2 ± 20.1 ^ab^	<0.01
V	16.1 ± 29.3	18.8 ± 29.9	15 ± 28.8	0.08	2.6 ± 11.7 ^a^	7.9 ± 20.9 ^b^	1.1 ± 6.8 ^a^	<0.01	14 ± 22.9	13.6 ± 23.7	13.9 ± 24.6	0.912
XIII	13.2 ± 26.2 ^a^	14.6 ± 28 ^a^	20.2 ± 32.5 ^b^	<0.01	13.7 ± 26.2	17.2 ± 30.2	8.4 ± 23.1	<0.01	18.3 ± 26.5	18.9 ± 25.4	22 ± 29.1	0.1
VI	16.3 ± 29 ^a^	19.9 ± 30.2 ^b^	20.4 ± 31.5 ^b^	<0.01	7.7 ± 18.6 ^b^	8.1 ± 22.2 ^b^	3.6 ± 14.9 ^a^	<0.01	14 ± 22.7 ^a^	14.7 ± 23.4 ^a^	19.8 ± 28.4 ^b^	<0.01
VII	22.4 ± 28.4 ^a^	22.6 ± 31.4 ^a^	30.5 ± 34.8 ^b^	<0.01	18.6 ± 27.4 ^b^	24.1 ± 32.7 ^c^	11.7 ± 25.4 ^a^	<0.01	9.7 ± 18.5	11.1 ± 21.5	9.8 ± 20.8	0.06
VIII	37.2 ± 30 ^b^	33.8 ± 31.4 ^a^	36.2 ± 31.5 ^b^	<0.01	8.2 ± 18.1 ^b^	15.4 ± 25.2 ^c^	4.4 ± 14.2 ^a^	<0.01	5.1 ± 11.3 ^a^	10.5 ± 16.8 ^c^	8 ± 17.7 ^b^	<0.01
IX	46.8 ± 26.4 ^c^	38.3 ± 30.8 ^a^	42.4 ± 28.6 ^b^	<0.01	6 ± 16.3 ^b^	13 ± 23.5 ^c^	4.3 ± 13.3 ^a^	<0.01	4.4 ± 9.6 ^a^	7 ± 14.1 ^b^	4.5 ± 12.5 ^a^	<0.01
XIV	43.2 ± 30.1 ^b^	34.6 ± 32.1 ^a^	36.2 ± 32.1 ^a^	<0.01	16.6 ± 24.1 ^a^	28.9 ± 31.7 ^c^	24.2 ± 31.7 ^b^	<0.01	6.6 ± 13.6 ^b^	5 ± 12.1 ^a^	4.3 ± 12 ^a^	<0.01
X	30.3 ± 29.2 ^b^	28 ± 28.7 ^a^	32.8 ± 31.4 ^c^	<0.01	27.9 ± 29.7 ^b^	31.9 ± 30.1 ^c^	26.1 ± 31 ^a^	<0.01	4.6 ± 10.7 ^c^	3.6 ± 9.4 ^b^	2.9 ± 8.6 ^a^	0.11
XI	43.3 ± 26.8 ^c^	39 ± 25.9 ^b^	33.6 ± 28.3 ^a^	<0.01	4.3 ± 12 ^a^	9.3 ± 17.7 ^b^	5.9 ± 15 ^a^	<0.01	2.3 ± 8.8 ^a^	4 ± 11 ^b^	2.5 ± 9.4 ^a^	<0.01
XII	0 ± 0	63.7 ± 30.2	71.5 ± 30.5	0.36	0 ± 0	2.5 ± 7.6	1.9 ± 6.7	0.76	0 ± 0	0 ± 0.2	0.3 ± 1	0.12
Total	33.1 ± 30.7 ^b^	30.4 ± 31 ^a^	35.4 ± 31.5 ^c^	<0.01	14.4 ± 24.5 ^b^	22.3 ± 29.5 ^c^	11.9 ± 24.2 ^a^	<0.01	7.2 ± 15.4 ^b^	7.4 ± 15.9 ^b^	6.3 ± 15.9 ^a^	<0.01

^a,b,c^ Within rows, averages with different superscript differ significantly.

**Table 4:** Distribution of farms according to the herd size based on cow livestock units (CLUs) per farm.

Herd Size (CLU/Farm)	1997	2007	2021	Variation (1997–2021)
≤10	34,841 (74.29%) *	12,234 (63.45%) *	10,551 (74.19%) *	−69.7%
11–50	9273 (19.77%) *	5027 (26.07%) *	2553 (17.95%) *	−72.5%
51–200	2250 (4.8%) *	1380 (7.16%) *	631 (4.44%) *	−72.0%
201–500	473 (1.01%) *	522 (2.71%) *	296 (2.08%) *	−37.4%
501–1000	54 (0.12%) *	95 (0.49%) *	132 (0.93%) *	144.4%
>1000	9 (0.02%) *	22 (0.11%)	59 (0.41%) *	555.6%
Total	46,900 (100%)	19,280 (100%)	14,222 (100%)	−69.7%

* Cells where there is no independence between rows and columns.

**Table 5:** Stocking rate according to herd size based on cow livestock units (CLUs) per farm.

Herd Size (CLU/Farm)	1997	2007	2021	*p* Value
≤10	0.96 ± 2.33 ^a^	1.05 ± 1.93 ^b^	0.97 ± 1.68 ^a^	<0.01
11–50	0.92 ± 3.88 ^a^	1.09 ± 2.84 ^b^	1.58 ± 3.97 ^c^	<0.01
51–200	0.99 ± 2.69 ^a^	1.1 ± 0.85 ^a^	1.49 ± 3.08 ^b^	<0.01
201–500	1.09 ± 0.82 ^a^	1.29 ± 0.85 ^b^	1.45 ± 0.88 ^c^	<0.01
501–1000	1.12 ± 0.57	3.21 ± 17.93	2.43 ± 6.01	0.55
>1000	1.39 ± 0.95	1.5 ± 0.71	2.63 ± 7	0.66
Total	1.0 ± 2.7 ^a^	1.1 ± 2.5 ^b^	1.1 ± 2.4 ^b^	<0.01

^a,b,c^ Within rows, averages with different superscript differ significantly.

**Table 6:** Average herd size and number of farms according to stocking rate (bovine livestock unit, BLU/ha) categories.

	Herd Size (CLU)				Number of Farms				
Socking Rate (BLU/ha)	1997	2007	2021	*p* Value	1997	2007	2021	*p* Value	Variation (1997–2021)
Less than 1 BLU/ha	14 ± 65 ^b^	21 ± 100 ^c^	14 ± 51 ^a^	0.00	35,470 (72.5%) *	12,679 (59.6%) *	9287 (57.2%) *	0.00	−73.8%
1–2 BLU/ha	26 ± 70 ^a^	52 ± 128 ^c^	84 ± 683 ^b^	0.00	7795 (15.9%) *	4807 (22.6%) *	3340 (20.6%) *		−57.2%
2–3 BLU/ha	15 ± 71 ^a^	34 ± 96 ^b^	61 ± 189 ^b^	0.00	1754 (3.6%) *	1012 (4.8%) *	869 (5.3%) *		−50.5%
3–4 BLU/ha	16 ± 64 ^a^	40 ± 187 ^ab^	44 ± 318 ^b^	0.05	626 (1.3%) *	273 (1.3%) *	302 (1.9%) *		−51.8%
4–5 BLU/ha	9 ± 22 ^a^	19 ± 63 ^ab^	40 ± 156 ^b^	0.00	339 (0.7%) *	153 (0.7%)	134 (0.8%) *		−60.5%
5–10 BLU/ha	9 ± 30 ^a^	16 ± 59 ^ab^	39 ± 189 ^b^	0.00	603 (1.2%)	238 (1.1%)	190 (1.2%)		−68.5%
More than 10 BLU/ha	8 ± 15 ^a^	16 ± 57 ^ab^	45 ± 164 ^b^	0.00	313 (0.6%)	118 (0.6%)	100 (0.6%)		−68.1%

^a,b,c^ Within rows, averages with different superscript differ significantly. * Cells where there is no independence between rows and columns.

**Table 7:** Natural pastures (% of farm area) according to bovine stocking rate category.

Bovine Stocking Rate (BLU/ha)	1997	2007	2021	*p* Value
Less than 1	36.3 ± 30 ^a^	33.7 ± 30.2 ^b^	36.6 ± 30.1 ^a^	<0.01
1–2	25.3 ± 30.8 ^a^	24.7 ± 31.1 ^a^	34.8 ± 33.6 ^b^	<0.01
2–3	21.3 ± 31.1 ^a^	24 ± 32.4 ^a^	30.5 ± 33.4 ^b^	<0.01
3–4	14.6 ± 27 ^a^	20.6 ± 31.1 ^a^	33.3 ± 36.1 ^b^	<0.01
4–5	15.9 ± 29.1 ^a^	23.1 ± 33 ^b^	23.8 ± 34 ^b^	<0.01
5–10	15.5 ± 29.6 ^a^	21.5 ± 33.2 ^a^	30.3 ± 36.2 ^b^	<0.01
More than 10	12.6 ± 28.9 ^a^	20.7 ± 33.6 ^ab^	18.8 ± 29.9 ^a^	<0.01
Total	33.1 ± 30.7 ^b^	30.4 ± 31 ^a^	35.4 ± 31.5 ^c^	<0.01

^a,b,c^ Within row, averages with different superscript differ significantly.

**Table 8:** Improved pasture (% of farm area) according to bovine stocking rate category.

Bovine Stocking Rate (BLU/ha)	1997	2007	2021	*p* Value
Less than 1	12.4 ± 22.1 ^b^	18.1 ± 26.2 ^b^	7.1 ± 17.5 ^a^	<0.01
1–2	23.9 ± 30.8 ^a^	33.8 ± 32.9 ^b^	22.4 ± 31.3 ^a^	<0.01
2–3	16.3 ± 28.3 ^a^	26.5 ± 33.5 ^b^	23.2 ± 33.7 ^b^	<0.01
3–4	13.8 ± 28.5 ^a^	21 ± 32.3 ^b^	15.6 ± 30.8 ^a^	<0.01
4–5	10.4 ± 25.6 ^a^	14.8 ± 31.1 ^ab^	16.9 ± 31.3 ^c^	0.05
5–10	8.9 ± 23.5 ^a^	13.3 ± 29.8	6.6 ± 21.8 ^a^	0.01
More than 10	7 ± 21.5 ^a^	10.4 ± 25 ^b^	2.8 ± 14.4 ^a^	0.01
Total	14.4 ± 24.5 ^a^	22.3 ± 29.5 ^b^	11.9 ± 24.2 ^a^	<0.01

^a,b,c^ Within row, averages with different superscript differ significantly.

**Table 9:** Sown pasture (% of farm area) according to bovine stocking rate.

Bovine Stocking Rate (BLU/ha)	1997	2007	2021	*p* Value
Less than 1	4.9 ± 11.1 ^b^	4.8 ± 11.5 ^b^	4.3 ± 12 ^a^	<0.01
1–2	12.6 ± 19.9 ^c^	11.5 ± 19.1 ^b^	8.9 ± 18.9 ^a^	<0.01
2–3	17.1 ± 26.5 ^c^	15 ± 24.5 ^a^	11.8 ± 22.9 ^a^	<0.01
3–4	20.8 ± 29.8 ^b^	16.9 ± 25.7 ^ab^	13.5 ± 26.3 ^a^	<0.01
4–5	14.8 ± 27.8	17.7 ± 29.6	11.7 ± 21.7	0.17
5–10	18.4 ± 30.5	14.7 ± 26.5	14.3 ± 27.6	0.12
More than 10	15.9 ± 29.5	10.7 ± 25.2	12 ± 27	0.18
Total	7.2 ± 15.4 ^b^	7.4 ± 15.9 ^b^	6.3 ± 15.9 ^a^	<0.01

^a,b,c^ Within row, averages with different superscript differ significantly.

**Table 10:** Definition of variables and calculation formula.

Variable	Abbreviation	Description	Calculation Formula
Bulls	Bulls	Number of bulls in the herd	
Cows	CLU	Livestock units corresponding to cows	
Size herd	SH	Bovine livestock units	
Total bovine	NBT	Number of bovines	
Total livestock units	TLU	Livestock units of all animal species on the farm	
Farm area	Farm area	Total farm area	
Relative presence of cows in the herd	CLUB	Percentage of bovine livestock units that correspond to cows	$\mathrm{CLUB}=\frac{CLU}{BLU}$
Relative presence of cows in the farm	CLUT	Percentage of total livestock units that correspond to cows	$\mathrm{CLUT}=\frac{CLU}{TLU}$
Bovine stocking rate	BSR	Bovine stocking rate	$\mathrm{BST}=\frac{CLU}{Farm\;area}$
Farm stocking rate	FST	Farm stocking rate	$\mathrm{FSR}=\frac{TLU}{Farm\;area}$
Relative presence of natural pasture	RPNP	Percentage of the property area occupied by natural grassland	$\mathrm{RPNP}=\frac{Natural\;pasture\;area}{Farm\;area}\times 100$
Relative presence of improved pasture	RPIP	Percentage of the property area occupied by improved grassland	$\mathrm{RPIP}=\frac{Improved\;pasture\;area}{Farm\;area}\times 100$
Relative presence of sown pasture	RPSP	Percentage of the property area occupied by planted meadow	$\mathrm{RPSP}=\frac{Sown\;pasture\;area}{Farm\;area}\times 100$
Relative presence of forest species	RPF	Percentage of the property area occupied by forest species	$\mathrm{RPF}=\frac{Forest\;area}{Farm\;area}\times 100$
Relative presence of orchard	RPO	Percentage of the property area occupied by fruit species	$\mathrm{RPO}=\frac{Orchard\;area}{Farm\;area}\times 100$
Relative presence of crops	RPC	Percentage of the property area occupied by crops	$\mathrm{RPC}=\frac{Crops\;area}{Farm\;area}\times 100$

**Table 11:** Eigenvalues and explained variance.

Component	Eigenvalues	% of Variance	% Cumulative
1	3.438	34.4	34.4
2	1.885	18.9	53.3
3	1.255	12.6	65.9
4	1.1	11	76.9

**Table 12:** Rotated component matrix.

	Principal Component			
Variable	1	2	3	4
Bulls	0.705	−0.065	0.031	−0.025
Cows	0.945	0.137	0.073	0.042
CLUB	−0.051	0.908	0.034	0.093
CLUT	0.105	0.904	0.11	0.071
SH	0.971	0.033	0.076	0.04
BSR	−0.028	0.07	−0.016	0.621
TLU	0.967	0.008	0.06	0.038
RPNP	−0.044	−0.01	−0.802	−0.382
RPIP	0.123	0.141	0.864	−0.199
SPP	0.089	0.059	0.087	0.812

**Table 13:** Number and percentage of farms per census and group.

Group	Farm Number (% of Farms)			Variation (1997–2021)
	1997	2007	2021	
I: Extensive System	16,168 (34.5%) *	7251 (37.6%)	7121 (50.1%) *	−9047 (−56.0)
II: Dual-purpose extensive system	16,193 (34.5%) *	4589 (23.8%) *	3923 (27.6%) *	−12,270 (−75.8%)
III: Specialized semi-intensive system	9101 (19.4%) *	5669 (29.4%) *	2056 (14.5%) *	−7045 (−77.4)
IV: Intensive system	5432 (11.6%) *	1771 (9.2%) *	1122 (7.9%) *	−4310 (−79.3)

* Cells where it is not possible to assert independence between census and group.

**Table 14:** Average variables by group.

Variables	Group I	Group II	Group III	Group IV	*p* Value
Bulls (number)	0.5 ± 2.3 ^a^	0.7 ± 12.3 ^a^	0.9 ± 2.3 ^b^	0.6 ± 1.4 ^a^	<0.01
Cows (LU)	18.8 ± 92.2 ^b^	8.5 ± 232.5 ^a^	42.6 ± 103.8 ^d^	36.6 ± 157.9 ^c^	<0.01
CLUB (%)	73 ± 13.7 ^d^	42.0 ± 14.0 ^a^	64.8 ± 15 ^b^	68.1 ± 16.3 ^c^	<0.01
CLUT (%)	57 ± 17.3 ^c^	27.8 ± 10.4 ^a^	55.0 ± 17.0 ^c^	52.7 ± 19.5 ^b^	<0.01
SH (bovine LU)	27.1 ± 122.8 ^b^	19.7 ± 332 ^a^	65.9 ± 155.1 ^d^	56.5 ± 247.5 ^c^	<0.01
TLU (total LU)	31.1 ± 125.5 ^a^	26.9 ± 335.4 ^a^	69.8 ± 157.5 ^c^	62.2 ± 261.1 ^b^	<0.01
Bovine stocking rate (bovine LU/ha)	0.8 ± 0.8 ^a^	0.6 ± 0.7 ^a^	1.0 ± 0.8	3.1 ± 6.5	<0.01
Farm stocking rate (total LU/ha)	1.0 ± 1.8 ^a^	1.0 ± 1.5 ^a^	1.3 ± 1.2 ^b^	4.6 ± 11 ^c^	<0.01
Total bovine (number)	36.6 ± 160.9 ^a^	27.7 ± 438.4 ^a^	91.5 ± 217.1 ^d^	78.3 ± 345.1 ^c^	<0.01
RPNP (%)	50.6 ± 29.6 ^d^	38.2 ± 27.2 ^c^	5.6 ± 10.2 ^a^	6.7 ± 14.3 ^a^	<0.01
RPIP (%)	3.8 ± 8.8 ^a^	4 ± 9.5 ^a^	59.8 ± 20.7 ^c^	6.3 ± 12.9 ^b^	<0.01
RPSP (%)	2.5 ± 5.7 ^a^	2.6 ± 6 ^a^	4.6 ± 7.5 ^b^	42.4 ± 24.9 ^c^	<0.01
RPF (%)	0.14 ± 0.21 ^b^	0.23 ± 0.26 ^c^	0.1 ± 0.14 ^b^	0.05 ± 0.11 ^a^	<0.01
RPO (%)	0.008 ± 0.044 ^b^	0.007 ± 0.039 ^a^	0.008 ± 0.027 ^b^	0.007 ± 0.044 ^ab^	<0.01
RPC (%)	0.15 ± 0.22 ^b^	0.16 ± 0.21 ^b^	0.12 ± 0.15 ^a^	0.23 ± 0.25 ^c^	<0.01
Farm area (ha)	72.9 ± 471.6 ^b^	82.2 ± 635.9 ^c^	72 ± 144.8 ^b^	50.1 ± 441.9 ^a^	<0.01

^a,b,c,d^ Within row, averages with different superscript differ significantly.

**Table 15:** Average variables by group and censuses.

Variable	Group I				Group II				Group III				Group IV			
	1997	2007	2021	*p* Value	1997	2007	2021	*p* Value	1997	2007	2021	*p* Value	1997	2007	2021	*p* Value
Bulls (number)	0.47 ± 1.14 ^a^	0.56 ± 1.48 ^b^	0.54 ± 4.27 ^b^	<0.01	0.55 ± 1.64 ^a^	0.86 ± 4.98 ^ab^	1.16 ± 30.19 ^b^	0.014	0.73 ± 1.35 ^a^	0.97 ± 2.32 ^b^	1.42 ± 4.31 ^c^	<0.01	0.53 ± 1.16 ^a^	0.71 ± 1.68 ^b^	0.65 ± 1.77 ^b^	<0.01
Cows (LU)	16 ± 45 ^a^	21 ± 67 ^b^	24 ± 165 ^b^	<0.01	7 ± 23	9 ± 52	15 ± 579	0.18	27 ± 56 ^a^	47 ± 99 ^b^	97 ± 208 ^c^	<0.01	24 ± 62 ^a^	59 ± 264 ^b^	59 ± 233 ^b^	<0.01
CLUB (%)	71.5 ± 12.7 ^a^	72 ± 13 ^b^	77.5 ± 15.4 ^c^	<0.01	41.4 ± 13.9 ^a^	44.3 ± 13.3 ^b^	41.6 ± 14.5 ^a^	<0.01	63.7 ± 14.7 ^a^	64.7 ± 15.1 ^b^	69.8 ± 15.5 ^c^	<0.01	67.3 ± 15.6 ^a^	68 ± 16.4 ^a^	72 ± 19 ^b^	<0.01
CLUT (%)	54.3 ± 16 ^a^	57.1 ± 16 ^b^	62.8 ± 19.8 ^c^	<0.01	27.3 ± 10.1 ^a^	29.4 ± 10.6 ^c^	28.2 ± 11.2 ^b^	<0.01	53 ± 16.1 ^a^	55.3 ± 17.1 ^b^	63.4 ± 18.1 ^c^	<0.01	51.6 ± 18.5 ^a^	54.4 ± 19.5 ^b^	55.4 ± 23.4 ^b^	<0.01
SH (bovine LU)	23.2 ± 68.4	30.8 ± 101.1	32.3 ± 208.8	<0.01	17.6 ± 58.5	21.4 ± 114.1	26.5 ± 815.5	0.304	44.4 ± 90.5 ^a^	74.2 ± 154.2 ^b^	138 ± 296.2 ^c^	<0.01	39.8 ± 106.1 ^a^	90.8 ± 429.8 ^b^	83.7 ± 323.7 ^b^	<0.01
TLU (total LU)	27.5 ± 73.5 ^a^	35 ± 104.9 ^b^	35.3 ± 209.9 ^b^	<0.01	24.7 ± 71.8	29.5 ± 125.8	33 ± 817.7	0.31	48.4 ± 93.8 ^a^	78.5 ± 157.4 ^b^	140.9 ± 297.5 ^c^	<0.01	44.9 ± 111.3	98.8 ± 461.2	88.4 ± 325.6	<0.01
Bovine stocking rate (bovine LU/ha)	0.69 ± 0.74 ^a^	0.83 ± 0.87 ^b^	0.86 ± 0.93 ^b^	<0.01	0.55 ± 0.63 ^a^	0.68 ± 0.81 ^b^	0.74 ± 0.86 ^c^	<0.01	0.94 ± 0.8 ^a^	1.08 ± 0.78 ^b^	1.33 ± 0.86 ^c^	<0.01	2.91 ± 5.93 ^a^	3.14 ± 7.43 ^a^	3.9 ± 7.61 ^b^	<0.01
Farm stocking rate (total LU/ha)	0.94 ± 1.29	1.1 ± 1.46	1.12±2.85	<0.01	0.89 ± 1.5 ^a^	1.07 ± 1.6 ^b^	1.16 ± 1.64 ^c^	<0.01	1.17 ± 1.21 ^a^	1.31 ± 1.11 ^b^	1.49 ± 1 ^c^	<0.01	4.38 ± 10.43	4.7 ± 11.34 ^a^	5.77 ± 12.85 ^b^	<0.01
Total bovine (number)	32 ± 95 ^a^	42 ± 142 ^b^	42 ± 264 ^b^	<0.01	25 ± 91	31 ± 166	36 ± 1069	0.304	62 ± 129 ^a^	104 ± 219 ^b^	187 ± 407 ^c^	<0.01	56 ± 155 ^a^	126 ± 599 ^b^	113 ± 439 ^b^	<0.01
RPNP (%)	51 ± 29.3 ^b^	51.8 ± 28.8 ^b^	48.6 ± 30.7 ^a^	<0.01	39.4 ± 27 ^c^	37.1 ± 27.3 ^b^	34.7 ± 27.6 ^a^	<0.01	6 ± 10.5 ^b^	5.1 ± 9.7 ^a^	5.1 ± 10.1 ^a^	<0.01	6.2 ± 13.6 ^a^	6.7 ± 14.7 ^a^	9.7 ± 16.7 ^b^	<0.01
RPIP (%)	3.5 ± 8.2 ^b^	5.1 ± 9.9 ^c^	3.2 ± 8.7 ^a^	<0.01	3.7 ± 9.4	5.7 ± 10.7	2.8 ± 8	<0.01	57.2 ± 21 ^a^	62.4 ± 19.9 ^b^	64.4 ± 19.9 ^c^	<0.01	6.4 ± 12.6 ^b^	7.9 ± 14.4 ^b^	3.2 ± 10.8 ^a^	<0.01
RPSP (%)	2.4 ± 5.2 ^a^	2.8 ± 6.2 ^b^	2.4 ± 6.1 ^a^	<0.01	2.4 ± 5.5 ^a^	3 ± 6.7 ^c^	2.8 ± 6.9 ^b^	<0.01	4.8 ± 7.3 ^b^	4.7 ± 8 ^b^	3.2 ± 6.7 ^a^	<0.01	39.5 ± 24.5 ^a^	46.8 ± 23.6 ^b^	49.1 ± 26.8 ^b^	<0.01
RPF (%)	0.13 ± 0.21 ^a^	0.13 ± 0.19 ^a^	0.15 ± 0.23 ^b^	<0.01	0.23 ± 0.26 ^b^	0.22 ± 0.24 ^a^	0.25 ± 0.27 ^c^	<0.01	0.1 ± 0.15 ^a^	0.1 ± 0.14 ^a^	0.11 ± 0.15 ^b^	<0.01	0.05 ± 0.11	0.04 ± 0.1	0.05 ± 0.12	0.625
RPO (%)	0.0085 ± 0.041 ^c^	0.0003 ± 0.011 ^b^	0.015 ± 0.065 ^a^	<0.01	0.0064 ± 0.0376 ^b^	0.0004 ± 0.0068 ^a^	0.0164 ± 0.0611 ^c^	<0.01	0.0129 ± 0.033 ^c^	0.0001 ± 0.0011 ^a^	0.0084 ± 0.0321 ^b^	<0.01	0.0054 ± 0.0295 ^b^	0.0004 ± 0.056 ^a^	0.0278 ± 0.0013 ^c^	<0.01
RPC (%)	0.19 ± 0.24 ^c^	0.13 ± 0.2 ^b^	0.09 ± 0.17 ^a^	<0.01	0.19 ± 0.22 ^c^	0.13 ± 0.2 ^b^	0.1 ± 0.17 ^a^	<0.01	0.15 ± 0.17 ^c^	0.09 ± 0.12 ^b^	0.04 ± 0.1 ^a^	<0.01	0.27 ± 0.25 ^c^	0.21 ± 0.23 ^b^	0.08 ± 0.16 ^a^	<0.01
Farm area (ha)	74.6 ± 336.8	73.1 ± 290.3	69 ± 781.4	0.71	83.1 ± 527.7 ^b^	96.5 ± 953.8 ^b^	61.9 ± 576.7 ^a^	0.42	59.8 ± 112.3 ^a^	77.9 ± 154.5 ^b^	110.3 ± 218.6 ^c^	<0.01	38.4 ± 101.5 ^a^	86.3 ± 927.4 ^b^	49.5 ± 197.3 ^a^	<0.01

^a,b,c^ Within row, averages with different superscript differ significantly.

## Data Availability

The data presented in this study are available on request from the corresponding author due to the size of the database and the online availability of the data at the following address: https://www.ine.gob.cl/estadisticas/economia/agricultura-agroindustria-y-pesca/censos-agropecuarios.

## Appendix A

**Table A1:** Regional distribution of the groups.

	1997					2007					2021				
Region	Group I	Group II	Group III	Group IV	*p* Value	Group I	Group II	Group III	Group IV	*p* Value	Group I	Group II	Group III	Group IV	*p* Value
XV	2 (5.7%) *	2 (5.7%) *	0 (0%) *	31 (88.6%) *	<0.01	0 (0%) *	11 (26.2%)	1 (2.4%) *	30 (71.4%) *	<0.01	3 (12%) *	2 (8%) *	0 (0%) *	20 (80%) *	<0.01
I	0 (0%)	0 (0%)	0 (0%)	6 (100%)		0 (0%)	1 (25%)	0 (0%)	3 (75%) *		2 (20%)	5 (50%)	0 (0%)	3 (30%) *	
II	1 (2.1%) *	0 (0%) *	2 (4.3%) *	44 (93.6%) *		0 (0%)	0 (0%)	0 (0%)	5 (100%) *		2 (14.3%) *	3 (21.4%)	0 (0%)	9 (64.3%) *	
III	16 (21.1%) *	32 (42.1%)	0 (0%) *	28 (36.8%) *		5 (20%)	6 (24%)	0 (0%) *	14 (56%) *		3 (8.3%) *	13 (36.1%)	0 (0%) *	20 (55.6%) *	
IV	254 (36.5%)	265 (38.1%)	16 (2.3%)	161 (23.1%)		89 (33.1%)	67 (24.9%)	44 (16.4%) *	69 (25.7%) *		99 (41.6%) *	75 (31.5%)	3 (1.3%) *	61 (25.6%) *	
V	575 (32.5%)	442 (25%) *	60 (3.4%) *	694 (39.2%) *		249 (39.3%)	143 (22.6%)	64 (10.1%) *	178 (28.1%) *		93 (41.7%) *	56 (25.1%)	5 (2.2%) *	69 (30.9%) *	
XIII	816 (30.5%) *	353 (13.2%)	497 (18.6%) *	1012 (37.8%) *		209 (32%) *	97 (14.8%) *	141 (21.6%) *	207 (31.7%) *		99 (39.4%) *	36 (14.3%) *	27 (10.8%)	89 (35.5%) *	
VI	924 (36.3%)	508 (19.9%) *	334 (13.1%) *	782 (30.7%) *		329 (45.2%) *	134 (18.4%) *	80 (11%) *	185 (25.4%) *		195 (47.2%)	78 (18.9%) *	17 (4.1%) *	123 (29.8%) *	
VII	1262 (29.8%) *	1152 (27.2%) *	1158 (27.4%) *	662 (15.6%) *		472 (35.9%)	230 (17.5%) *	435 (33.1%) *	179 (13.6%) *		567 (52.8%)	206 (19.2%) *	162 (15.1%)	138 (12.9%) *	
VIII	3016 (36.2%) *	3650 (43.8%) *	1004 (12.1%) *	654 (7.9%) *		1065 (42.8%) *	565 (22.7%)	511 (20.5%) *	346 (13.9%) *		1292 (59%) *	579 (26.5%)	101 (4.6%) *	216 (9.9%) *	
IX	3182 (37.5%) *	4317 (50.8%) *	624 (7.3%) *	367 (4.3%) *		1338 (42.5%) *	1047 (33.2%) *	553 (17.6%) *	211 (6.7%) *		2689 (57.5%) *	1587 (33.9%) *	187 (4%) *	216 (4.6%) *	
XIV	2652 (41.9%) *	1952 (30.8%)	1265 (20%) *	463 (7.3%) *		1351 (37.5%)	811 (22.5%) *	1274 (35.4%) *	165 (4.6%) *		832 (41.2%) *	539 (26.7%)	561 (27.8%) *	87 (4.3%) *	
X	3093 (28.3%) *	3212 (29.4%) *	4122 (37.7%) *	511 (4.7%) *		1842 (31.9%) *	1253 (21.7%) *	2521 (43.6%) *	162 (2.8%) *		1096 (39%) *	671 (23.9%) *	977 (34.8%) *	63 (2.2%) *	
XI	375 (52.2%) *	308 (42.8%) *	19 (2.6%) *	17 (2.4%) *		271 (49.9%) *	210 (38.7%) *	45 (8.3%) *	17 (3.1%) *		136 (59.6%) *	69 (30.3%)	16 (7%) *	7 (3.1%) *	
XII	---	---	---	---		31 (68.9%) *	14 (31.1%)	0 (0%) *	0 (0%) *		13 (72.2%)	4 (22.2%)	0 (0%)	1 (5.6%)	

* Cells where it is not possible to assert independence between region and group.
